# Qualitative Findings From a Survey Measuring Informational Needs and Quality of Life of Women Living With Metastatic Breast Cancer

**DOI:** 10.1002/pon.70177

**Published:** 2025-05-10

**Authors:** Rachel Starkings, Lesley Fallowfield, Stephanie Russ, Valerie Jenkins

**Affiliations:** ^1^ SHORE‐C Brighton and Sussex Medical School University of Brighton and University of Sussex Brighton UK

**Keywords:** breast cancer, cancer, coping, metastatic, oncology, qualitative, quality of life

## Abstract

**Background:**

People living with metastatic breast cancer (MBC) may have different support requirements to those with early stage breast cancer (EBC). These differences can be substantial, particularly as care pathways and information are often designed around the latter. There is limited understanding of how these discrepancies impact patients with MBC.

**Aims:**

In the LIMBER study (Living with Metastatic Breast Cancer), we explored the distinct and unmet needs of people living with MBC.

**Methods:**

In collaboration with people living with MBC and healthcare professionals (HCPs), we developed an online survey comprising fixed and free text responses. Fixed responses and overall study demographics from the main LIMBER study have been published elsewhere. A framework analysis of the free text comments is reported here.

**Results:**

The resulting thematic map has seven main themes ‐ friends and family, reactions of others, healthcare professionals, systems & processes, knowledge & information, outlook & goals and wellbeing. Participants reflected that comments made by friends and family were often well‐meaning but showed misunderstanding of the disease. This was particularly noticeable in understanding the difference between MBC and EBC. There were references to the lack of support and information from HCPs.

**Conclusions:**

The analysis of free text comments from this survey demonstrates the impact that MBC can have, particularly without robust support or accessible information. Understanding areas where patients have outstanding needs provides insight into how best to promote coping strategies and improved quality of life, while informing those who provide informal and formal care.

## 1 Background

Diagnosis of metastatic breast cancer (MBC) is increasing both globally and within the UK [1, 2]. Advances to treatments for this group have improved overall prognosis [2, 3, 4]. While MBC is not curable, good supportive care can improve quality of life outcomes for patients [5]. In comparison, our understanding of patients' experiences of the disease, information provision and impact of different care pathways remains limited [6, 7, 8].

There are distinct demands for those with MBC which differ from those of patients living with early stage breast cancer (EBC) [3, 6, 7, 9, 10]. These can be physical, practical or emotional, impacting the ability of patients to cope [7, 11, 12]. In the MBC setting where cure is not an objective, realistic goals and lifestyle considerations are important and demand recognition from family, friends and HCPs [5, 13, 14, 15, 16]. Studies reveal considerable support and information gaps for those with MBC [7, 15] with increased rates of anxiety and depression compared to patients in the EBC setting [17, 18]. Despite the benefits from improved treatments, psychological coping and adjustment have important links to increased survival and improved quality of life as well [9, 19, 20, 21]. Ensuring that these areas are addressed is therefore important.

The health‐based theory of coping model outlines how people living with a health condition navigate the impact of disease [22]. It acknowledges that good coping mechanisms do not aim to put distance between patient and prognosis, but allow the individual to focus on managing associated emotional and practical impacts [22]. As MBC is incurable, the primary aim for patients is to sustain meaningful wellbeing while addressing value‐based goals rather than trying to find strategies which reframe or minimize that reality [16].

Alongside psychological wellbeing, there is equal emphasis on professional and social interactions as contributing to quality of life [10, 22, 23]. In order to ameliorate outstanding needs for patients living with MBC, there is often a requirement for additional support, be it from healthcare professionals (HCPs) or friends and family members [24, 25]. For example, HCPs can provide relevant clinical information, but are also looked to for hope or guidance [11, 12, 13]. This interplay between emotional and information need can be hard to deliver.

A workshop for patients and HCPs from 27 different countries held during the 2019 Advanced Breast Cancer meeting (ABC19) identified several areas of concern [26]. With variability in healthcare systems, and patient circumstances, we explored unmet needs specific to the UK in the LIMBER (Living with Metastatic Breast Cancer) study. The online survey captured people's experiences of MBC, communication, information, understanding of treatment, quality of life and support from family, friends and HCPs. Participants were prompted to write things that were helpful and those which could be improved. Here we present a qualitative analysis of the free text comments provided.

## 2 Methods

An online semi‐structured questionnaire was developed with input from HCPs and patients with lived experience of the disease. The survey comprised a mix of fixed and free responses along with demographic items. Following a process of refinement and user testing, the questionnaire was hosted for 3 months on the SHORE‐C website. The project was funded by the charity, Make 2nds Count and received ethical approval from the research governance and ethics committee as part of Brighton and Sussex Medical School (ref: ER/RMLS21/7). Participants were self‐selecting and all completed an online consent form before participating. Full details of the survey and its development have been published elsewhere, along with the main results [3].

### 2.1 Qualitative Analysis

There were 10 free‐text questions included in the survey which we explored via a framework approach to thematic analysis within an interpretivist paradigm. Due to the nature of using free text questions as part of the survey, answers focused on how individuals interpreted their own experiences. We in turn saw codebook development as interpretive rather than applying fixed categories and ideas to the data [21]. The more open ended, constructionist view was not possible with this dataset.

These were transposed into a grid and uploaded into NVivo software. There was no link between individuals and comments to limit any bias. The researchers were blinded to the identification of participants and comments were read thematically by question rather than reviewing individual cases. All comments were read independently by two coders (R.S., S.R.). After initial reading, each coder made a provisional list of emergent themes from the material. These lists were reviewed for overlap and divergence. A common list of themes was created, forming a provisional codebook. This was applied independently by the two coders to three of the free text questions. Coding was merged and compared, finding over 80% agreement. Any disagreements were reviewed and the codebook was amended further to reflect changes and include illustrative examples.

The remaining seven free text questions were split between the two coders. Coding was merged together and themes were divided between the coders to review coherence and thematic agreement. Queries were discussed and changes made as necessary.

This initial coding was reviewed by two individuals living with MBC. They provided feedback on any discrepancies, overall coherence and relevance to the population. Once there was consensus between the research team and PPI members, a thematic map was developed, grouping themes and sub‐themes together. This was reviewed for agreement by three researchers (R.S., S.R., V.J.).

## 3 Results

**FIGURE 1:**
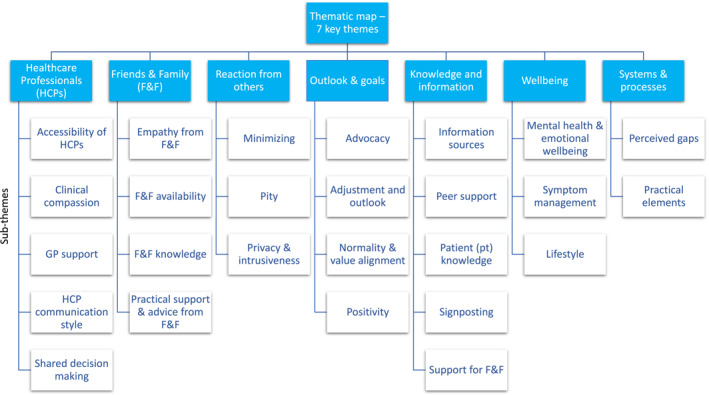
LIMBER Thematic map.

Of the 143 people completing the LIMBER survey, 136 provided anonymous responses to the free‐text questions. Demographics for these individuals are provided in Table 1. Full study demographics are available elsewhere [3]. Following analysis, the resulting thematic map had 7 themes ‐ friends & family (F&F), reactions from others, healthcare professionals (HCPs), systems & processes, knowledge & information, outlook & goals, and wellbeing. Each theme had associated sub‐themes, see Figure 1 for the thematic map.

**TABLE 1:** Demographics.

*N* = 136	
Age	
Range	28–77
Mean (SD)	51.7 (9.3)
Age groups	
25–34	5 (4%)
35–44	28 (21%)
44–54	1 (< 1%)
45–54	50 (37%)
55–64	41 (30%)
65–74	10 (7%)
75+	1 (< 1%)
Ethnicity	
White	124 (91%)
Other	12 (9%)
Highest education	
None	3
GCSE/A‐levels	45
Vocational	20
Undergraduate degree or higher	68
Employment status	
Not working	92 (68%)
Working	44 (32%)
Main support	
Spouse	95 (70%)
Child	9 (7%)
Parent	3 (2%)
Sibling	7 (5%)
Friend	17 (12%)
Other/none	5 (4%)
How long have you lived with MBC?	
< 1 year	42 (31%)
1–2 years	43 (32%)
2–5 years	31 (23%)
> 5 years	20 (15%)
First diagnosis of primary or metastatic cancer?	
Primary	93 (68%)
Metastatic	43 (32%)
Lines of treatment	
1	80 (59%)
2	19 (14%)
3	12 (9%)
4+	15 (11%)
None or unknown	10 (7%)
Current treatment(s)^*^	
Chemotherapy	45 (33%)
Endocrine therapy	67 (49%)
Immunotherapy	4 (3%)
Radiotherapy	12 (9%)
Surgery	10 (7%)
Targeted therapy	77 (57%)
Other (e.g. clinical trial) or none	19 (14%)

* Participants could be on multiple ongoing treatments.

### 3.1 Friends, Family and the Reactions of Others

A main source of support for participants was their friends and family members. They described a range of practical tasks these groups undertook, alleviating some additional pressure and logistical burdens.

They walked the dog when I couldn't. Drove me to appointments. Took me for drives when I was unable to get out. Collected shopping for me. Raised money for us as a family to make memories together.

Alongside these pragmatic responses, respondents also reflected on the emotional care and support offered by these networks. This ranged from taking time to listen, providing a space for someone to be honest about their feelings, consistently checking in, or doing things to show solidarity.

They called me, messaged me or came round to distract me and cheer me up. They held me when I was distraught and didn't want to cry in front of children. They sent flowers every time I had chemo.

More striking though was an apparent lack of awareness about the impact comments and suggestions could have on the patient. Participants largely referred to their diagnosis being “ignored” or dismissed. Family and friends offered suggestions, such as making dietary changes or remaining upbeat, imploring the individual to be “positive”.

Suggest I give up dairy, eat bitter almond kernels, have an alkaline diet etc and I’d be cured!

Without a better understanding of MBC, some friends and family members tried to reframe the situation, urging the patient to consider mortality phlegmatically, providing context that uncertainty exists for everyone, with or without a terminal diagnosis.

Being told that “I could be hit by a bus tomorrow none of know how long we have got!!”

These platitudes were often seen by respondents as an indication that friends and family members struggled to know what to say or do. People in wider social circles also failed to appreciate the nature of the disease or react appropriately, minimizing the situation or asking too many questions. This left respondents with a sense of isolation, unable to receive meaningful and informed support from those they may have wanted to turn to.

Some friends haven’t known what to say or what to do so they just disappeared. Saying things like “you can beat this” (no, no I can’t, it’s terminal, it’s never going away) it’s hard making people realise that it’s never going away.

### 3.2 Healthcare Professionals, Systems and Processes

Respondents left text about the compassion and accessibility of healthcare professionals. The tone of these interactions ranged from supportive to feeling like a nuisance. There were equally varied reports about the accessibility of clinical help, again with some participants feeling informed with proactive engagement from HCPs, while others felt in the dark, unsure who to even contact.

I have no named nurse and I only see a consultant quarterly. Since my diagnosis I have seen 4 different consultants or interns. This leaves me feeling that no‐one in the NHS is in the least bit invested in my treatment or well‐being. I find this very depressing.

The implication of this communication was that participants either felt emotionally supported, and safe, or isolated and precarious.

My oncologist was very caring, open minded, has always had my back. The best thing he did at my first meeting with him, when I was still in shock and grief at the dx, was to give me his mobile number.

They appear to be so busy, that when I walk away from consultations with my oncologist I don’t feel reassured, or as if they have my back. It’s like they detach emotionally to survive the difficult job that they do, but that leaves me with a disconnect from my oncologist. My life is in their hands, but I feel that I am just a number/ a workload to them which scares me. As I feel the attention to detail is lacking, which feels dangerous where stage 4 cancer is concerned.

Some of these difficulties in accessing services extended to the primary care setting. Participant feedback highlighted a perceived lack of awareness of, or initiative to investigate, signs of recurrence for those who had a prior early stage diagnosis.

GP didn't listen understand or investigate my extreme pain before I was diagnosed despite having primary breast cancer 3 years before.

Some of this tension wasn't aimed at clinical individuals directly but rather the processes involved. Systems seemed too rigid for some, such as patients who continued to work and needed their appointment schedule to be flexible to accommodate this. Others spoke of their frustration at arriving on time for appointments only to wait hours to be seen. Participants remarked that services weren't always synchronized, leading to a knock‐on effect due to delays. Clinical reports were sometimes unavailable or contained incorrect information, causing alarm.

Reports are sloppy: my last started with a statement that I had had my left kidney removed. How a transcriber can confuse breast with kidney, I don't know, but my oncologist read the report to me this time so we were able to rectify the major error.

### 3.3 Knowledge and Information

Participants reflected that the nature of MBC is misunderstood by people, either not knowing it exists or assuming there are various treatment options available as there are in the EBC setting. There was a desire for information to be made accessible and available to people offering them support.

Yes, I wish there was an info sheet explaining the illness and types if treatment and encouraging people to stick around for social and emotional support. I feel very alone at times

It was apparent that participants didn't feel that the burden of responsibility to educate the public should remain with the patient. They spoke about a wish for people to undertake their own research and understand the implications of the disease.

How difficult it is for friends to understand living with secondary cancer. I may not be helping by using that term!? My family get it/ they've been at face to face sessions, listened to recordings and, in lockdown, shared in the telephone consultations‐ (unforeseen bonus). Other people haven't got used to the notion that metastatic disease isn't necessarily rapidly terminal now, anything that assists public understanding, without making heroines of us, and without us having to do it all ourselves, would be really helpful.

This lack of information for caregivers was also apparent for patients who themselves were left confused about the disease. Those who had a previous primary diagnosis weren't always aware that a metastatic diagnosis was possible, beyond a recurrence in the breast.

I wish I'd known about MBC full stop. Had no idea it existed. I thought if it came back it would be just in the breast.

There was additional confusion for those who had never received a primary breast cancer diagnosis, but were instead diagnosed with MBC in the first instance. People spoke about not knowing this was a possibility and not understanding the differences.

I didn't know you could be diagnosed metastatic as a first diagnosis. I was diagnosed de novo.

There was a further lack of understanding about what the signs and symptoms of a recurrence were. Participants commented that, as they didn't know the signs, they felt uninformed to monitor these.

After 10 years since primary breast cancer diagnosis, I thought that I had “won the cancer battle”. When I started losing weight, feeling extremely tired with a swollen tummy it never crossed my mind that breast cancer might be the cause.

Even after diagnosis there was uncertainty about the treatment and prognosis for an MBC diagnosis. This could refer to the ordering of treatment or available lines of therapy. Participants also felt they were lacking knowledge about other things they could do to help themselves, be it diet, exercise or complimentary therapies.

Would love to hear more about nutrition but no one is interested in this area and I think it's a massive opportunity being missed.

To overcome these knowledge gaps, some participants were given specific information relating to MBC or were referred to services such as Maggie's, counseling, local cancer centers, or palliative care teams. Others were left to seek out support and education on their own. Regardless of clinical information shared, participants spoke about the importance of peer engagement. One of the strongest recommendations from participant comments was how useful online community groups were to find others in the same position.

Make 2nds count website Facebook group and Instagram has been a huge help. I have made friends with women like me from all over the UK that understand what it is like with MBC. You don’t feel alone and everyone is on their journey but routing for you on yours. It’s a truly wonderful community.

Another clear finding was to avoid blanket searches online. Instead, participants spoke about the value of using reputable websites and referring any questions or uncertainties to the oncology team instead of the internet. They felt unreliable or old information was too readily available which could then become frightening for people.

Do NOT use Google. Do NOT use Google. I can't repeat it enough.

### 3.4 Outlook and Wellbeing

Facing variable levels of support from loved ones and healthcare professionals, participants reflected on their own mental adjustment to the diagnosis. Some left text about using time to get their affairs in order, others noted that having a treatment plan in place returned some control to them.

The waiting for a treatment plan is horrible ‐ once you get that plan in place things will improve and you will start to gain a little control again. Look after yourself, try not to make any major decisions until you're ready.

Participants reported that their emotional wellbeing was often overlooked. While it was recognized that they experienced physical symptoms of MBC, mental repercussions were ignored. This meant that the psychological impact of the disease was often managed without clinical support.

I have never been asked how I am emotionally. Clinics seem too busy and health professionals don’t have the time to talk. Just the physical pain and symptoms are dealt with.

Largely there was a sense that people varied in the time needed time to adapt to the diagnosis and focus on what felt important. Participants urged others to take one step at a time and not rush ahead.

There is life, it will be shorter and different but you have more left

Respondents outlined a balance between being realistic about the facts while maintaining a positive sense of the future. Participants spoke about being the ones who should dictate this authentically, rather than being told to “be positive”.

Some I thought felt that I did not fully understand prognosis but I did but just I wanted some hope. So, they felt it was their duty to make me understand situation fully without asking what I understood & how I wanted to approach the prognosis which was accepting, pragmatic but hopeful that treatment will manage disease

There was a desire to be treated as normal. Participants did not want extra attention or pity due to their diagnosis, but instead wanted their loved ones to engage with them in the same ways they had prior to MBC. Some individuals wanted the clinical system to also recognize their humanity, without reducing them to a diagnosis.

Anything to make me feel like a human being, an individual—and not just a body being processed through a system

Participants valued maintaining elements of their life that brought them joy, anything from walking the dog to traveling. Some discussed the steps their clinical teams were taking to help them achieve this.

How important it is for me to have pain relief so I can walk my dogs and get some fresh air

An example of this stemmed from managing side effects which impact lifestyle and relationships. Sexual wellbeing in particular was disregarded, yet individuals wanted more information about their libido.

The whole picture of my health they only want to talk about the side effects of treatment I would like to talk to someone about my libido for example.

## 4 Discussion

Striking in our findings was the lack of knowledge patients and their friends and family had about MBC and its implications. Information needs are high in this cohort, particularly as the disease progresses [20]. Not understanding that MBC could exist, as we saw in the survey, poses an area for early psychoeducational intervention. Research indicates improved self‐efficacy when people are provided with training as to what signs and symptoms of recurrence are, and how to monitor them. Information materials can be created alongside patient feedback and delivered early to improve awareness [27]. This provision could ameliorate concerns identified in other research that the perception of limited resources enables a sense of isolation in patients [8].

The lack of awareness about MBC extended to friends and family. As noted in other research, the prominent battle language around cancer is not appropriate in the MBC setting [24]. Yet, this can still often be used by those supporting patients with the disease. Suggestions that other lines of treatment will be available, or that cancer had been overcome once so it could be done again, demonstrate a misunderstanding of what a metastatic diagnosis means compared to EBC.

The relationship with healthcare professionals is key to adjustment for any cancer treatment, but particularly in the metastatic setting [12, 13]. Tailored communication to meet patient need and explore treatment options and outcomes with quality of life in mind, can improve someone's sense of efficacy and foster shared decision making [11, 14]. Yet, text from this survey indicates that the patient/professional relationship is variable. Without good communication and accessibility, patients can feel isolated and vulnerable [12]. Comments highlighted a lack of understanding of the disease in a primary setting and a lack of consistency in secondary care. When respondents endorsed their clinical support, it was often related to the accessibility of a named person and shared discussions centered on what was meaningful for the patient.

To address these gaps in support we developed two information films, one for friends and family members and one for HCPs [3]. Using survey quotes, we created character vignettes to highlight some of the key challenges we identified. These materials are aimed at ameliorating some of the misunderstandings or missed opportunities participants directly shared with us. The video designed for friends and family members is available online (https://tinyurl.com/ycknfcny) and an educational/communication training program for HCPs is currently is under development.

Alongside education about the disease itself, LIMBER respondents wanted additional information about its wider impacts. There were particular concerns identified, such as sexual intimacy, which largely felt overlooked but remained important for some participants. This is in keeping with literature suggesting there are areas of life which are impacted but which go unmentioned, leaving patients unprepared [16, 28, 29].

Participants also referenced their emotional wellbeing and coping strategies. They discussed optimism and reframing techniques, but were clear that the use of these should be governed by the individual, not imposed upon them. Coping styles present an opportunity for intervention and can be indicative of quality of life outcomes. The areas of need identified in this survey map to both the emotional and practical realms of coping [19, 22]. The need to attune to both of these speaks to the pervasive intrusion MBC has on patient wellbeing [30].

Within the social cognitive processing theory, both uncertainty and avoidance are correlates to psychological distress [23]. Mediating these relationships are interpersonal factors such as support from HCPs and informal caregivers [23]. For example, there is evidence to suggest that dyadic and individual coping is improved when both partners use similar strategies [25]. The misunderstanding and misalignment discussed in this study could then impinge on the ability of friends and family to appropriately help individuals adapt to a diagnosis, while also impacting their own wellbeing.

Evident in our results, people with a diagnosis of MBC want further information and clarity about diagnosis, treatments and prognosis. However, they also want to be in control of the emotional appraisal of their situation. As suggested in other research, the interplay of the emotional and knowledge based aspects can impact patient outcomes and sense of efficacy [10]. Further work could be done to explore these domains, providing additional avenues for intervention to manage distress and its subsequent correlates to survival [21].

### 4.1 Clinical Implications

The findings from the LIMBER survey, and particularly the free text comments, indicate a disconnect between patient need and information provision. Clinically, there is room to improve on communication about the disease – that it exists, what patients should look for, and signs of recurrence. There is also further work needed to improve the communication of healthcare professionals in addressing what is most important to individual patients. The survey was initially designed to capture any unmet needs in the MBC setting broadly. As such, we did not differentiate our qualitative results based on demographics and clinical details. The themes we captured were present across responses, which tells us about the overall experience of this cohort. The range of treatment experiences seen in the respondents suggests there is an opportunity to further explore unmet needs relevant to stage at diagnosis, treatment type, line of treatment etc. This would allow for more precise information and support to be offered.

### 4.2 Study Limitations

This study does have its limitations. Namely, this was a self‐selecting sample who found the survey opportunity via a cancer charity website. Responders might have had more polarized experiences than those who did not respond. As with any qualitative analysis, there is an acknowledgment that not every experience can be accounted for. However, having a large sample means we had a variety of comments which mediated any outlying experiences.

Our population included those who were initially diagnosed with early stage disease and those who presented de novo. There was also variability in time since diagnosis, lines of treatment and current treatment. However, our sample was largely homogenous in ethnicity. More work is needed to reach marginalized communities who may have different experiences of this disease.

Additionally, we must consider that an online‐only survey does marginalize those without internet access. A paper copy was available to be completed but we did not receive any requests for this. As this was completed once, there are no follow up timepoints to see if issues were ameliorated over time and as it was self‐completed, we cannot ask any follow up questions or clarifications.

## 5 Conclusions

Qualitative results from this survey indicate wide reaching impacts from MBC across social, informational and emotional domains. Understanding these support needs allows us to consider areas for improved communication and tools to better equip patients to cope with the disease. By providing a better infrastructure to patients, they may be afforded better self‐efficacy. More work is needed to develop tools for patients but also their friends, family and healthcare professionals.

## Author Contributions

L.F. and V.J. developed and designed the LIMBER study. R.S. and S.R. led on qualitative analysis and framework development. The first draft of the manuscript was written by R.S. and all authors provided feedback on subsequent versions, approving the final submission.

## Conflicts of Interest

The authors declare no conflicts of interest.

## Data Availability

The data that support the findings of this study are available from the corresponding author upon reasonable request.

## References

[1] C. Palmieri , J. Owide , and K. Fryer , “Estimated Prevalence of Metastatic Breast Cancer in England, 2016‐2021,” JAMA Network Open 5, no. 12 (2022): e2248069, 10.1001/jamanetworkopen.2022.48069. 36547985 PMC9857429

[2] F. Cardoso , D. Spence , S. Mertz , et al., “Global Analysis of Advanced/Metastatic Breast Cancer: Decade Report (2005–2015),” Breast 39 (2018): 131–138, 10.1016/j.breast.2018.03.002. 29679849

[3] L. Fallowfield , R. Starkings , C. Palmieri , et al., “Living With Metastatic Breast Cancer (LIMBER): Experiences, Quality of Life, Gaps in Information, Care and Support of Patients in the UK,” Supportive Care in Cancer 31, no. 8 (2023): 459, 10.1007/s00520-023-07928-8. 37432501 PMC10335945

[4] A. Welt , S. Bogner , M. Arendt , et al., “Improved Survival in Metastatic Breast Cancer: Results from a 20‐Year Study Involving 1033 Women Treated at a Single Comprehensive Cancer Center,” Journal of Cancer Research and Clinical Oncology 146, no. 6 (2020): 1559–1566, 10.1007/s00432-020-03184-z. 32189107 PMC7230039

[5] F. Cardoso , N. Wilking , R. Bernardini , et al., “A Multi‐Stakeholder Approach in Optimising Patients’ Needs in the Benefit Assessment Process of New Metastatic Breast Cancer Treatments,” Breast 52 (2020): 78–87, 10.1016/j.breast.2020.04.011. 32450470 PMC7487948

[6] S. Lewis , K. Willis , J. Yee , and S. Kilbreath , “Living Well? Strategies Used by Women Living With Metastatic Breast Cancer,” Qualitative Health Research 26, no. 9 (2016): 1167–1179, 10.1177/1049732315591787. 26130655

[7] T. Lyons‐Rahilly , P. Meskell , E. Carey , E. Meade , D. O’ Sullivan , and A. Coffey , “Exploring the Experiences of Women Living With Metastatic Breast Cancer [MBC]: A Systematic Review of Qualitative Evidence,” PLoS One 19, no. 1 (2024): e0296384, 10.1371/journal.pone.0296384. 38181009 PMC10769043

[8] E. Kemp , B. Koczwara , P. Butow , et al., “Online Information and Support Needs of Women With Advanced Breast Cancer: A Qualitative Analysis,” Supportive Care in Cancer 26, no. 10 (2018): 3489–3496, 10.1007/s00520-018-4206-1. 29693203

[9] D. Keane , G. Phillips , N. Mitchell , R. M. Connolly , and J. Hegarty , “Improving Quality of Life and Symptom Experience in Patients With Metastatic Breast Cancer: A Systematic Review of Supportive Care Interventions,” Psycho‐Oncology 32, no. 8 (2023): 1192–1207, 10.1002/pon.6183. 37434307

[10] S. Alfieri , L. Murru , M. Bosisio , et al., “‘Ariadne’s Thread’: Psycho‐Educational Empowerment Intervention for Patients With Metastatic Breast Cancer,” Journal of Cancer Education 39, no. 6 (2024): 663–680, 10.1007/s13187-024-02449-2. 38809494

[11] M. M. Vila , Sd Barco Berron , M. Gil‐Gil , C. Ochoa‐Arnedo , and R. V. Vázquez , “Psychosocial Aspects and Life Project Disruption in Young Women Diagnosed With Metastatic Hormone‐Sensitive HER2‐Negative Breast Cancer,” Breast 53 (2020): 44–50, 10.1016/j.breast.2020.06.007. 32623094 PMC7375669

[12] M. Franklin , S. Lewis , J. Townsend , M. Warren , F. Boyle , and A. L. Smith , “Making the Unbearable, Bearable: Qualitative Examination of Patient, Family and Nurses’ Perspectives on the Role and Value of Specialist Metastatic Breast Care Nurses,” European Journal of Oncology Nursing 69 (2024): 102523, 10.1016/j.ejon.2024.102523. 38342058

[13] K. A. Bland , R. Mustafa , and H. McTaggart‐Cowan , “Patient Preferences in Metastatic Breast Cancer Care: A Scoping Review,” Cancers 15, no. 17 (2023): 4331, 10.3390/cancers15174331. 37686607 PMC10486914

[14] G. B. Rocque , A. Rasool , B. R. Williams , et al., “What Is Important When Making Treatment Decisions in Metastatic Breast Cancer? A Qualitative Analysis of Decision‐Making in Patients and Oncologists,” Oncologist 24, no. 10 (2019): 1313–1321, 10.1634/theoncologist.2018-0711. 30872466 PMC6795158

[15] M. E. Clarijs , J. Thurell , F. Kühn , et al., “Measuring Quality of Life Using Patient‐Reported Outcomes in Real‐World Metastatic Breast Cancer Patients: The Need for a Standardized Approach,” Cancers 13, no. 10 (2021): 2308, 10.3390/cancers13102308. 34065805 PMC8151772

[16] A. C. Ginter , “‘The Day You Lose Your Hope is the Day You Start to Die’: Quality of Life Measured by Young Women With Metastatic Breast Cancer,” Journal of Psychosocial Oncology 38, no. 4 (2020): 418–434, 10.1080/07347332.2020.1715523. 32067600

[17] C. Iseki , “The Process of Reaching Psychological Adjustment Among Adult Women Diagnosed With Metastatic Breast Cancer and Receiving Cancer Pharmacotherapy,” Asia‐Pacific Journal of Oncology Nursing 10, no. 3 (2023): 100184, 10.1016/j.apjon.2023.100184. 36844250 PMC9944287

[18] B. Mossman , L. M. Perry , L. E. Walsh , et al., “Anxiety, Depression, and End‐Of‐Life Care Utilization in Adults With Metastatic Cancer,” Psycho‐Oncology 30, no. 11 (2021): 1876–1883, 10.1002/pon.5754. 34157174

[19] B. S. Noriega Esquives , E. A. Walsh , F. J. Penedo , et al., “Coping Strategies and Psychosocial Resources Among Women Living With Metastatic Breast Cancer: A Qualitative Study,” Journal of Psychosocial Oncology 42, no. 3 (2023): 1–17, 10.1080/07347332.2023.2254754. 37698184 PMC10927610

[20] S. Aranda , P. Schofield , L. Weih , et al., “Mapping the Quality of Life and Unmet Needs of Urban Women With Metastatic Breast Cancer,” European Journal of Cancer Care 14, no. 3 (2005): 211–222, 10.1111/j.1365-2354.2005.00541.x. 15952965

[21] S. L. Brown , J. F. Roush , A. J. Marshall , C. Jones , and C. Key , “The Intervening Roles of Psychological Inflexibility and Functional Impairment in the Relation Between Cancer‐Related Pain and Psychological Distress,” International Journal of Behavioral Medicine 27, no. 1 (2020): 100–107, 10.1007/s12529-019-09838-8. 31898310

[22] H. M. Stallman , “Health Theory of Coping,” Australian Psychologist 55, no. 4 (2020): 295–306, 10.1111/ap.12465.

[23] A. A. Panjwani , A. J. Applebaum , T. A. Revenson , J. Erblich , and B. Rosenfeld , “Intolerance of Uncertainty, Experiential Avoidance, and Trust in Physician: A Moderated Mediation Analysis of Emotional Distress in Advanced Cancer,” Journal of Behavioral Medicine 47, no. 1 (2024): 71–81, 10.1007/s10865-023-00419-5. 37285106 PMC10942744

[24] A. Guité‐Verret and M. Vachon , “The Incurable Metastatic Breast Cancer Experience through Metaphors: The Fight and the Unveiling,” International Journal of Qualitative Studies on Health and Well‐Being 16, no. 1 (2021): 1971597, 10.1080/17482631.2021.1971597. 34455941 PMC8409930

[25] W. Liu , F. M. Lewis , M. Oxford , and I. Kantrowitz‐Gordon , “Common Dyadic Coping and its Congruence in Couples Facing Breast Cancer: The Impact on Couples' Psychological Distress,” Psycho‐Oncology 33, no. 3 (2024): e6314, 10.1002/pon.6314. 38459736

[26] L. Fallowfield , F. M. Boyle , L. Travado , et al., “Gaps in Care and Support for Patients With Advanced Breast Cancer: A Report From the Advanced Breast Cancer Global Alliance,” JCO Global Oncology, no. 7 (2021): 976–984, 10.1200/go.21.00045. 34156869 PMC8457864

[27] A. Michaelides and C. Constantinou , “Integration of Longitudinal Psychoeducation Programmes During the Phases of Diagnosis, Management and Survivorship of Breast Cancer Patients: A Narrative Review,” Journal of Cancer Policy 23 (2020): 100214, 10.1016/j.jcpo.2019.100214.

[28] S. I. McClelland , “‘I Wish I'd Known’: Patients' Suggestions for Supporting Sexual Quality of Life After Diagnosis With Metastatic Breast Cancer,” Sexual and Relationship Therapy 31, no. 4 (2016): 414–431, 10.1080/14681994.2015.1093615.

[29] J. B. Reese , L. A. Zimmaro , S. McIlhenny , et al., “Coping With Changes to Sex and Intimacy After a Diagnosis of Metastatic Breast Cancer: Results From a Qualitative Investigation With Patients and Partners,” Frontiers in Psychology 13 (2022), 10.3389/fpsyg.2022.864893. PMC9019080 35465532

[30] M. A. Hoffman , R. W. Lent , and T. L. Raque‐Bogdan , “A Social Cognitive Perspective on Coping With Cancer: Theory, Research, and Intervention,” Counseling Psychologist 41, no. 2 (2013): 240–267, 10.1177/0011000012461378.

